# Undergraduate Nursing Students' Experiences of Virtual Learning during the COVID-19 Pandemic: A Qualitative Study

**DOI:** 10.1155/2024/7801500

**Published:** 2024-05-15

**Authors:** Zahra Asgari Tapeh, Azar Darvishpour

**Affiliations:** ^1^Department of Nursing, Faculty of Nursing and Midwifery, Iran University of Medical Sciences, Tehran, Iran; ^2^Department of Nursing, Zeyinab (P.B.U.H) School of Nursing and Midwifery, Guilan University of Medical Sciences, Rasht, Iran; ^3^Social Determinants of Health (SDH) Research Center, Guilan University of Medical Sciences, Rasht, Iran

## Abstract

**Background:**

With the emergence of the COVID-19 pandemic, schools and universities were closed, and virtual education replaced face-to-face classes. This learning method was a new and different experience for nursing students. Perceiving their experiences could help improve the quality of medical education. Therefore, the present study aimed to describe nursing students' experiences of virtual learning during the COVID-19 pandemic.

**Materials and Methods:**

This study involved qualitative descriptive research that was conducted in 2022. The participants included 25 undergraduate nursing students studying at the School of Nursing and Midwifery in East Guilan in northern Iran who had experienced virtual learning due to the COVID-19 pandemic. Purposeful sampling was applied until data saturation. Qualitative content analysis with a conventional approach was performed based on the model proposed by Graneheim and Lundman (2004). Coding was performed with MAXQDA 2007 software.

**Results:**

The data analysis led to the emergence of 110 primary codes and two main categories entitled “positive experiences” and “negative experiences.” The first main category was “positive experiences” (included 1 subcategory (benefits of virtual learning) with 3 subsubcategories (saving time, saving money, and increasing the possibility of daily planning)). The second main category was “negative experiences” (included 4 subcategories (reducing quality of education, physical effects, psychological effects, and different exams)).

**Conclusion:**

Nursing students had both positive and negative experiences with virtual learning during the COVID-19 pandemic and were facing multiple educational challenges. The findings of this study could be considered by managers and relevant officials in educational planning to improve the quality of nursing education.

## 1. Background

The first reported COVID-19 outbreak occurred in China in December 2019. The World Health Organization declared COVID-19 a pandemic on March 11, 2020 [1]. One month later, two confirmed cases were reported in Iran on February 19, 2020 [2]. The COVID-19 pandemic has had significant impacts on students' life and work, as well as their social life, financial status, and emotional health [3], and has led to numerous educational challenges [4]. This pandemic has also affected future visions of education [5]. Naturally, the pandemic has influenced nursing education [4]. A large number of countries have used quarantine and isolation as methods of treating this disease [6]. Many businesses and organizations were locked down at the beginning of this pandemic, and higher education institutions were no exception [4]. Educational institutions face a greater risk of nCoV spread due to crowded classrooms and strict protective measures [7]. This condition enabled education reforms focused on emerging technology [4]. In Iran, with the rapid spread of COVID-19, the Ministry of Health and Medical Education established policies to limit the further spread of this infection. The decision was made to replace face-to-face classes with virtual education [2, 8]. It is necessary to mention that virtual education, which can involve offline or online learning activities, was first offered offline, after which online education was added [9, 10]. Various virtual education software and systems are utilized globally, including spaces for sharing materials, dialogs, discussions, surveys, exams, and reporting [11, 12]. During the COVID-19 pandemic, Iranian medical universities used Navid software, developed for university learning and supported by the Smart University of Medical Sciences [11].

Online medical education is not new, and studies worldwide confirm the effectiveness of e-learning, which is widely adopted by learners globally [13, 14]. However, considering the acceptance of e-learning by students as a crucial factor contributing to the success of e-learning systems within the realm of education technology [9], during the COVID-19 pandemic, virtual learning replaced traditional classrooms, created a unique and compulsory experience for students, and left no alternate routes for education [15]. Research on virtual education during the COVID-19 pandemic has shown that students were primarily concerned about the impact of COVID-19 on medical education [16], and a major impact of the pandemic is the extension of academic terms [17]. Despite these educational challenges, this exceptional situation provides a great opportunity for nursing students to learn, take initiative, and be creative [18]. Although e-learning was crucial for education during the COVID-19 pandemic, Iranian medical students favor face-to-face or hybrid (mixed online and on-site) courses [19] for improved knowledge, skills, and social ability [9]. Tabatabai et al. discussed the impact of COVID-19 on medical education, virtual medical education, and the promotion of digital learning. They stated that new innovative strategies need to be used in the medical education system to support continuous education and assessment [20, 21]. Online education offers advantages over traditional methods, but monotonous monologs, lack of participation, logistical issues, and repetitive tasks in classes can cause boredom [22, 23]. As the unanticipated transition from face-to-face education to virtual education was challenging for students and educators in Iran, it is important to analyze the benefits, obstacles, potential changes, and development plans of online education from the perspective of medical students and educators [24, 25]. In addition, nursing students had to address global health issues [9], and their theoretical and clinical education was disrupted unexpectedly by the COVID-19 pandemic [4]. Despite the importance of nursing students' experiences with virtual learning, which could help improve the structure and use of online education in nursing, there are few reports on how COVID-19 has impacted nursing students' experiences with online learning. However, in some quantitative studies, nursing students' satisfaction with the quality of courses and virtual learning during the COVID-19 pandemic has been investigated [26, 27], but fewer qualitative studies have been conducted in this area. Therefore, this study aimed to describe nursing students' experiences with virtual learning during the COVID-19 pandemic.

## 2. Materials and Methods

### 2.1. Study Design and Participants

This study involved qualitative descriptive research that was conducted in 2022. Qualitative research is the best method for exploring the ideas and values of various groups [28].

The participants included 25 undergraduate nursing students studying at the Zeyinab (P.B.U.H.) School of Nursing and Midwifery in East Guilan in northern Iran who had experienced virtual learning due to the COVID-19 pandemic. A homogeneous sampling technique was used in the present study. Homogenous sampling is a purposive sampling technique that aims to achieve a homogeneous sample. This technique is commonly used when the research topic is specific to the features of a particular population [29].

The selection of participants was based on the inclusion criteria, and the sampling was continued until data saturation. The inclusion criteria included studying for a bachelor's degree in nursing, having a history of at least one semester of virtual learning, and having rich experiences and the ability to communicate and express experiences. The data were saturated with 22 students, and 3 others were interviewed for more certainty.

### 2.2. Data Collection

This study was approved by the ethics committee of Guilan University of Medical Sciences (Approval Number: IR.GUMS.REC.1399.598). The aim of the study was explained to all of the participants. Written informed consent was obtained from the participants. The participants were assured that they could withdraw from the research whenever they wished, and all their information would remain confidential.

Semistructured interviews were conducted in an in-depth manner to gather information. The duration of each interview was 30–45 minutes. The interviews were conducted individually in one of the classes of the nursing faculty.

Interviews began with questions such as “What is your experience of online learning during the quarantine due to the COVID-19 pandemic?”, “What are the main differences between online and attendance classes?”, “What factors are affecting your learning during the COVID-19 pandemic?”, “What are the advantages and disadvantages of virtual learning?”, “What are the barriers to virtual learning?”, and “Would you like the virtual learning to be continued?” There were also exploratory and follow-up questions such as “What do you mean?”, “'How?”, and “Can you explain more about that?” for further collection of details. The researcher used field notes to record observations, interactions, communication, and nonverbal gestures. Each interview was recorded with the participant's permission, implemented verbatim, and then analyzed on the same day. The data collection lasted 5 months.

### 2.3. Data Analysis

The data were analyzed via conventional qualitative content analysis following the recommendations of Graneheim and Lundman [30]. Content analysis, as a valid research method for data analysis [31], is a systematic and purposeful way to describe a phenomenon [32]. Qualitative content analysis is a common method used in nursing studies and is suitable for various contexts and data [33]. By using coding and classification of data techniques, this type of analysis aims to discover large quantities of textual information and examine its patterns, relationships, structures, and communication discourses [34].

For the data analysis, the transcripts were read several times. The identification of meaningful units was performed by reviewing all the participant descriptions and statements. Related sentences and expressions are underlined, and each significant phrase is assigned a code. In this way, meaning units were identified. Then, the meaning units were compressed. The codes were summarized and categorized according to their similarities and differences, after which the main categories emerged. The data were analyzed with MAXQDA 2007 software.

### 2.4. Ethics

The study was approved by the Ethical Committee of the Guilan University of Medical Sciences. Participation in the interview was completely voluntary. The participants were informed that the interview would be recorded and that the data collected would not be disclosed. Written informed consent was obtained from the participants. The participants were assured that they could withdraw from the research whenever they wished and that all their information would remain confidential.

### 2.5. Rigor

Rigor in qualitative analysis belongs to the process and its trustworthiness. It is essential for researchers to “immerse” themselves in data to explore all the possible nuances and relationships. Trustworthiness is considered a more appropriate criterion for evaluating qualitative studies [35].

In qualitative research, the concepts of credibility, dependability, and transferability have been used to describe various aspects of trustworthiness. Credibility deals with the focus of the research and refers to confidence in how well the data and processes of analysis address the intended focus [30]. There are many strategies for addressing credibility, including “prolonged engagement” and member checks [35]. In the present research, the most suitable method for collecting the data and the amount of data used were chosen, the participants were carefully chosen, their long-term contact with them was encouraged, and their trust was gained. Additionally, researchers' engagement with the data and continuous comparisons were used to ensure credibility.

Dependability is the degree to which data change over time and alterations are made in the researcher's decisions during the analysis process [30]. This process was described in sufficient detail to help another researcher repeat the work [35]. To ensure dependability, continuous reviews and comparisons of the data and categories were performed in terms of similarities and differences. Researchers referred to the findings and extracted codes to the participants for confirmation or correction. The authors also checked the findings with experts and informed researchers.

As with quantitative research, qualitative inquiry seeks to expand understanding by transferring findings from one context to another [36]. Transferability refers to the extent to which the findings can be transferred to other settings or groups [30]. Because qualitative research is specific to a particular context, a “thick description” of the particular research context is important because it allows the reader to assess whether it is transferable to their situation [35]. To meet these criteria, the researcher tried to provide detailed and rich descriptions of the research for the readers.

## 3. Results

Among the 25 participants, 16 were female and 9 were male, ranging in age from 20 to 24 years. In terms of years of education, 9 students were in their fourth year, 12 were in their third year, and 4 were in their second year.

**Figure 1:**
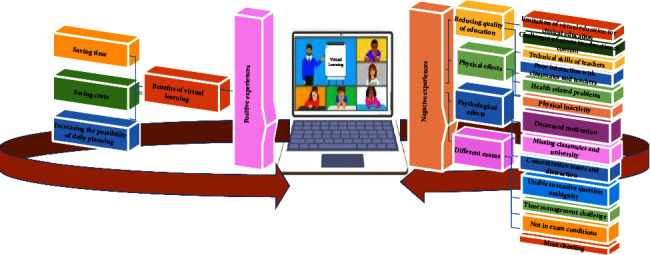
Diagram of the main categories and subcategories.

The data analysis led to the emergence of 110 primary codes and two main categories, “positive experiences” and “negative experiences,” with 5 subcategories and 15 sub-subcategories, which are described in the following section. A diagram of the main categories and subcategories is shown in Figure 1.  Table 1 shows how to form the first main category, entitled “Positive experiences.”

### 3.1. First Category: Positive Experiences

This category included 1 subcategory entitled “*benefits of virtual learning*” with 3 sub-subcategories (saving time, saving costs, and increasing the possibility of daily planning), which are explained as follows.

#### 3.1.1. Benefits of Virtual Learning

Participants stated that their most positive and effective experience with virtual education during the COVID-19 pandemic was that the education process became simpler and easier; in other words, it made it easier for them to learn lessons. This subcategory included 3 subsubcategories entitled “saving time,” “saving costs,” and “increasing the possibility of daily planning,” which are explained as follows.

During interviews, students repeatedly highlighted the effectiveness of online training in reducing the amount of time they spent receiving instruction on the same topic compared with in-person classes. This time includes class time, which is shorter in virtual classrooms, and travel time to the university, which is eliminated in online classes.

“In the online class, if something special happens to the internet or the online system of the university, the online classes start and end on time. Interestingly, all the teachers taught faster in online classes.”(P9, a 21-year-old female)

“It is true that in the face-to-face class, we may have more fun with our friends, but the truth is that the deviant discussions in the class or the interclass breaks make the class time longer, while teachers always complain that they were not able to teach the desired amount of lessons.” (P3, a 24-year-old male)

“I used to go to university 3 times a week; in fact, I'm on the road for 4 hours a week to get from home to school or vice versa. There is always traffic, but since the virtual classes are started, the total time for these courses could be 4 hours, which is great.” (P1, a 23-year-old female)

The participants highlighted the cost-reducing impact of online education. According to them, online education reduces financial burdens and saves money, especially during the COVID-19 pandemic, by eliminating travel, food, books, and other costs.

“The COVID-19 pandemic has hurt everyone financially, except students. The participants were asked to go to college, order food weekly, copy professors' handouts, and spend money with friends. All of this has been taken away by COVID-19.” (P5, a 22-year-old male)

“In my opinion, one of the main benefits of COVID-19, at least for me, is the reduction in transportation costs to and from college.” (P10, a 22-year-old female)

Participants stressed daily planning when discussing the benefits of saving time and money. Students reported that online education saves time and energy compared with face-to-face education, giving them more opportunities to plan and carry out daily personal affairs.

“During this pandemic, I have been able to practice guitars again. I just had the time and motivation and adjusted my schedule during the day to practice between online classes. It makes me feel better.” (P11, a 25-year-old female)

“You're home after the class, which is a very important feature. Because you have enough energy to perform your favorite activities after the class.” (P25, a 23-year-old male)

### 3.2. Second Category: Negative Experiences

This category included 4 subcategories entitled “*reducing the quality of education*,” “*physical effects*,” “*psychological effects*,” and “*different exams*,” which are explained as follows.

#### 3.2.1. Reducing the Quality of Education

Despite some positive experiences, online education in Iran has also presented challenges and negative experiences for nursing students. One of the negative experiences of the participants with virtual education was the decrease in quality of education, which was identified in 4 subsubcategories entitled “limitation of virtual education for clinical education,” “challenges of accessing educational content,” “technical skills of teachers,” and “poor interaction with classmates and teachers.” In the following, we will describe these issues.

Participants welcomed the opportunity to be away from hospital environments due to concerns about contracting and transmitting COVID-19 to their family members. However, they were concerned about the impact of being away from actual clinical fields such as hospitals on their learning. They considered it a lost opportunity to gain knowledge and practical skills about different treatments and procedures. They emphasized that although virtual education can cover theoretical education, the applications of digital technology in clinical education are limited, which means that it cannot replace clinical education. In their opinion, this negative experience impacted the quality of nursing education in their academic course during the COVID-19 pandemic. Some of the participants' statements in this regard are stated as follows:

“No matter how many photos, videos, slides, and theoretical training we see, we still have to see patients up close and perform procedures in person to know what is going on.” (P10, a 22-year-old female)

“I enjoy clinical internships. The pandemic was an exception, and reducing the time to attend the internship.” (P5, a 22-year-old male)

“The quality of our learning in this pandemic period was different from that of the students in previous courses. Virtual rounds and the presentation of cases in virtual form, etc., cannot replace clinical training. It was not a pleasant experience at all.” (P17, a 24-year-old female)

Students mentioned the challenges of accessing educational content as another factor affecting the quality of virtual education. Access to educational content for students depended on factors such as teachers' workloads, upload and download speeds, and university systems, which posed challenges. The participants said that virtual education heavily depends on teachers accessing educational content. Teachers sometimes did not realize that they should upload content on time, and students might wait until the end of the semester to make them available. This problem wastes time learning and increases exam stress. The situation was even worse for students who enrolled in courses without online classes and who received training entirely through videos prepared by their teachers. Additionally, the speed of the Internet affects not only students' participation in online classes but also their access to the content. Some students had high-speed Internet, but some students complained about Internet problems, dropping and reconnecting online, and slow download speeds. Sometimes, the system introduced by the university has technical issues, causing problems for teachers and students. Some of the participants' statements are mentioned as follows:

“Some professors are not interested in uploading the teaching files on time, and for online classes that are not saved, we have to make a screen record or take pictures during the class because we know that we do not have any files to read until the end of the semester.” (P15, a 22-year-old male)

“In the face-to-face class, most of the professors gave us the PowerPoint slides of that session after each class session, but this is not the case in the online class. I do not know what the reason is. Perhaps uploading files is difficult or time-consuming. After several sessions, they upload files, which is not very useful for students.” (P21, a 23-year-old female)

“When they give us the educational content that has been taught for several months at the end of the semester, how should we read them for the exam? One of the professors uploaded the educational files just a few days before the exam.” (P12, a 23-year-old female)

“Most of the time, the education system is fine, but sometimes it is annoying. Like when it kicks us out and we cannot get in, or when the teacher disconnects. Until you want to report the problem to the IT official and follow up, you have practically missed that class.” (P10, a 22-year-old female)

“For a quality online class, high-speed internet in an area with a strong signal is necessary.” (P1, a 23-year-old female)

Another factor that negatively affects the quality of online education according to the participants is its dependence on the technical skills of teachers. Students believe that some teachers are not suitable for online education and that the quality of their instruction is better in face-to-face classes. Some teachers' inability to create engaging audio and video content for online courses highlights their lack of preparedness for teaching in a virtual classroom, resulting in their one-way lectures in online classes.

Students say that teachers are classified into three groups: teachers reliant on blackboards and able to teach only in person; skilled teachers adept at creating engaging slide content with sound and images and capable of effectively teaching online; and teachers who teach similarly in person and virtually.

“All professors are not suited for the online class. For a professor whose face-to-face class lasted at least 2 hours, his/her virtual class hardly reached 45 minutes. Why? Because he/she cannot produce distance education content. This is to the detriment of students. Especially when it is about an important lesson.” (P13, a 22-year-old male)

Participants mentioned poor interaction with classmates and teachers in virtual classes as a negative factor affecting the quality of education. Online class communication happens only through a chat space on the page and is dependent on the teacher's attention on student comments and questions. The possibility of audio and video communication also exists, depending on the teacher's permission. This problem negatively affects students' interaction with their professor, ability to ask or answer questions, ability to solve problems, ability to interact with classmates, ability to create harmony in class, and, ultimately, the quality of education. In other words, in virtual education, group education is not induced, and education is individual.

“In face-to-face classes, when we have problems, the teacher solves them immediately, but in online classes, no matter how much we write on the chat box, he/she either does not see or read it until the end of the class. Therefore, he/she often does not have time to answer.” (P6, a 22-year-old female)

“Despite initially being glad that face-to-face classes had closed due to virtual classes, now I prefer being in the class atmosphere, sitting next to my friends and seeing the whiteboard and hearing the professors” voices up close for education.” (P11, a 25-year-old female)

#### 3.2.2. Physical Effects

Repeated use of mobile devices has led to health problems and inactivity in students, raising mental concerns for both students and parents. While students have standard classroom seating, there is no such space in the virtual classroom. Most participants complained of neck pain, eye fatigue, and headache after attending several hours of online classes. The students emphasized eye fatigue more. However, before and after face-to-face classes, students were more active on the university campus, which was lessened in virtual classes due to being at home. This subcategory included 2 subsubcategories (health problems and physical inactivity). Some of the participants' statements in this regard are stated as follows:

“I tend to spend a lot of time on my phone. Since classes have gone virtually, I spend most of the day looking at my phone in bed or chair, and lately, I feel like my eyes are tired or weak.” (P1, a 23-year-old female)

“Seating at the laptop table for hours causes neck pain and headaches despite changing positions frequently.” (P19, a 23-year-old female)

#### 3.2.3. Psychological Effects

In addition to its physical impact, online education had significant psychological effects. Decreased motivation, missing classmates and universities, and concentration issues and distractions were emphasized by the students during the interviews. As the quality of online teaching depends on several factors, some virtual classrooms are boring because of the teacher's teaching style, the slow speed of the online teaching platform, and the problems associated with uploading files; consequently, students are not motivated to attend class. This subcategory included 3 subsubcategories (decreased motivation, missing classmates and the university, and concentration issues and distraction). Some of the participants' statements in this regard are stated as follows:

“The frequent sound interruptions are annoying, causing me to avoid class” (P7, a 23-year-old female)

“Some teachers” classes are so monotonous that I fall asleep.” (P15, a 22-year-old male)

One of the limitations of virtual education for students is the lack of communication with classmates and teachers, as well as the distance from the classroom and university environment. This made them feel like they were missing from university and class, socializing with their classmates, friends, and teachers. This issue may affect students' social skills, competition, academic progress, and self-esteem.

“I miss the pleasure of seeing classmates, joking, eating together, and walking on the beautiful campus of the university.” (P9, a 21-year-old female)

The challenge of student concentration and distractions in virtual classes is a significant psychological effect of online education. Participants attributed this to the possibility of doing several tasks during online classes, for example, chatting, browsing, watching movies, talking with family at home, and eating.

“I cannot concentrate on the class and stare at the screen; I have to play with my phone at the same time and check it all the time” (P24, a 24\3-year-old male)

“Unfortunately, my family does not care at all that I am in an online class. There is constant movement in my room, which distracts me.” (P23, a 22-year-old male)

#### 3.2.4. Different Exams

Face-to-face and online education differs not only in the teaching process but also in the process of evaluating learners and exams. In face-to-face education, exam papers are provided to students, and they write the answers on the exam paper. During online education, students took exams using a virtual system provided by the university. The participants viewed the questions and then marked the answers or typed the descriptive answers within the allotted time. Virtual and in-person exams posed major challenges, mostly negative for participants. Differences included being unable to resolve question ambiguity, time management challenges, not being in exam conditions, and more cheating. In-person exams have the course teacher briefly available to address student inquiries. During the virtual exam, students could not clarify any ambiguity or speak to the professor, which was a downside. This subcategory included 3 sub-subcategories (unable to resolve question ambiguity, time management challenge, not in exam conditions, and cheating). Some of the participants' statements in this regard are mentioned as follows:

“In the exam of one of the courses, 2 questions were out of the book, and we could not do anything about it; we had to answer it because time was running out.” (P4, a 21-year-old female)

“Some questions are ambiguous, and it is not possible to understand the meaning. The professors should spend a few minutes and clear the ambiguities.” (P16, a 21-year-old female)

Students also objected to virtual exam timing. Exam times vary based on teachers' opinions, which are often set less than students' expectations. Despite the Internet's speed being a factor in virtual exams, descriptive virtual exams require typing, and students have different typing speeds. Therefore, VTE stress increased due to time management issues. However, on classic exams, students had more time to answer the questions.

“Teachers consider the exam time to be very short to prevent cheating, while in a face-to-face test, we never ran out of time.” (P10, a 22-year-old female)

“Rather than trying to answer the exam questions thoroughly, I was trying to do it as quickly as possible so that I would finish the exam on time.” (P1, a 23-year-old female)

In-person exams included an entrance card, assigned seating, preexam conversations, and silent answer writing. However, on the online exam, students wait at home to log in to the exam page through their computer, mobile, or tablet. The interviews revealed that home conditions differ from exam settings, causing distractions such as traffic, noise, and other factors that lead to students deviating from exam conditions during virtual exams.

“My room and closed door did not block out the noise from inside and outside the house or on the streets. My concentration was completely disrupted, making it feel like I was not even taking an exam” (P7, a 23-year-old female).

Some of the participants stated that they cheated during the virtual exams and were satisfied with the process of holding the exams online. However, some of the students were displeased by others cheating and considered the virtual exam and the resulting scores to be incorrect and unfair. The possibility of finding answers from books and pamphlets, chatting, and sharing answers were among the most important issues mentioned under the title of cheating in interviews. However, during face-to-face examinations, cheats are more difficult due to the presence of several invigilators and the prohibition of mobile phones, smartwatches, and other audio systems.

“Virtual training has caused a strange situation where students with previously low grades are now achieving high grades on exams. This puts those who genuinely study at a disadvantage” (P12, a 23-year-old female).

“The truth is that during the exams, we had a small group that shared the answers in the test exams. I do not think it is a bad thing. The exam should not be a giant. It is important to understand the lesson during the semester.” (P13, a 22-year-old male)

## 4. Discussion

The present study was conducted to describe nursing students' experiences with virtual learning during the COVID-19 pandemic. The nursing students' experiences led to the emergence of two main categories entitled “positive experiences” and “negative experiences.” Participants described that virtual training was associated with saving time and costs and improving the ability to plan and manage their daily schedules. Multiple studies, including that of Przymuszała et al., have shown that online classes are highly preferred by medical and health students in Poland. This preference stems from the convenience of not having to commute and the ability to utilize time more efficiently [37]. Additionally, one study in Iran demonstrated that virtual education and exams are especially advantageous during the COVID-19 pandemic, as they offer flexibility and accessibility while accommodating more students and saving resources [17]. Dental students in Taiwan have also found online education to be effective for time management and convenience [38]. However, in Dung's research at Hong Bang International University, students faced difficulties following study schedules and lacked self-discipline while taking online courses [39].

The second main category, titled “negative experiences,” included 4 subcategories entitled “reducing the quality of education,” “physical effects,” “psychological effects,” and “different exams.” From the point of view of students, the limitations of virtual education in clinical education, limited access to online content, limited technical skills, and poor interaction with classmates and instructors can reduce the quality of nursing education. The participants in the present study believed that virtual education could not provide sufficient support for clinical education, especially during crises such as the COVID-19 pandemic, where face-to-face education would be limited. In other words, when the pandemic shortened clinical training, virtual education was not able to compensate for this problem. These findings are consistent with previous research. For instance, Farsi et al. reported that nursing students, instructors, and administrators in Iran emphasized the importance of continuing nursing education, even in the face of challenges such as the COVID-19 pandemic and disruptions to clinical and blended learning [4]. Similarly, through a large-scale online survey across the world, Aristovnik et al. highlighted the need for online educators to ensure that professors possess the necessary knowledge and skills for virtual instruction and have the appropriate ICT equipment [3]. Distance learning studies in Israel and Turkey have also emphasized the importance of teacher-learner interactions in promoting learning, yet virtual education poses challenges because instructors lack nonverbal feedback [40, 41]. Goodwin et al. in Ireland reported that challenging online interactions reduced the quality of learning for nursing students [42]. However, the results of the present study were inconsistent with the results of De Ponti et al.'s study conducted on 122 sixth-year medical students in Portugal [43]. This difference in the findings may be related to the type of application of virtual education in the clinical setting; the use of virtual education in De Ponti et al.'s study involved patient introduction, case-based education, and rethinking. The purpose of the participants in the current study was to learn about clinical procedures that must be performed on real patients, and virtual training cannot be a suitable substitute for these procedures.

The second subcategory of the second main category (negative experiences) was the *physical effects* of online education. The spread of COVID-19 has caused changes in people's lifestyles [44]. While staying at home is considered a safe action, it may have unintended negative consequences because it leads to a decrease in physical activity [45]. The prevalence of obesity and a reduction in the body's immune system have increased due to home quarantine and the need to stay at home [44]. A lack of physical activity due to quarantine weakens the body's immune system, which leads to physical and mental health problems [46]. In China, Li and Che declared that online classes can cause physical symptoms such as head, neck, and eye pain, as well as muscle pain and inactivity, due to extended periods of online classes [47]. Additionally, a study in China showed that learners may experience fatigue and burnout, while parents worry about their children's health [48]. A study by Guo et al. in Canada reported that a sedentary lifestyle, along with fear and anxiety, impaired the physical health of many participants [46].

The third subcategory of the second main category (negative experiences) was the psychological effects of online education. Online education led to reduced motivation and disconnection from classmates and universities, resulting in feelings of missing friends and campuses. Concentration was difficult due to classroom distractions. According to Esra and Sevilen's study in Turkey, students' motivation for learning may decrease due to a lack of interaction, mismatched expectations and content, and difficulties organizing the learning environment. These results are consistent with those of other studies [49]. According to a survey about online learning, many students missed socializing with classmates [50]. A study by Goodwin et al. stated that distance education leads to distraction and inactivity in students [42]. In Martín-Sómer et al.'s study on student motivation during COVID-19 online classes in Spain, the majority believed that online education reduced learning and interest [51].

The fourth subcategory of the second main category (negative experiences) included the different exams. Students reported significant differences between virtual and in-person exams. Online exams had ambiguous questions, were harder to manage, and lacked exam conditions. Cheating was a major concern. These findings are consistent with the experiences of other groups from the virtual exam. Research in India has shown that online education presents challenges, including technical limitations, distractions, instructor competence, and learner well-being [52]. In their research in Oman, Sarrayrih and Ilyas mentioned that online tests require extensive preparation from both teachers and students. Student identification is crucial in online exams to prevent cheating and manage time. The system automatically logs out users after the allotted time and sends a warning [53]. In Yu-Fong Chang et al. study in Taiwan, dental students preferred traditional exams over online exams due to concerns about fairness and operational issues [38]. Abdelrahim in Bangladesh reported that during the COVID-19 pandemic, academic pressure, the ease of cheating, the desire to improve the grade point average, and the lack of monitoring led to increased cheating in virtual exams [54].

The COVID-19 pandemic has tremendously affected medical education and necessitated a dramatic pivot from in-person to virtual medical education. For example, online courses could be used as an alternative for students who cannot attend classes or benefit nursing students before they enter the clerkship stage; additionally, they could provide an opportunity for students to learn from the best lecturers in the world and expand their network for conducting research [20]. It is believed that all of the distresses caused by the COVID-19 pandemic can change e-learning practices in medical education forever [20]. However, special conditions and facilities are needed that could challenge medical educationists' ability to adapt to such unique situations [21].

Furthermore, the field of education is experiencing significant transformations, with advancements in medical sciences, technology, and educational tools emerging rapidly [5]. The speed of technological change will breakneck, creating high pressure to transform educational practices, institutions, and policies [55]. Many digital resources are now available for administrative and pedagogical support and enhancement in higher education. Today's students expect modern universities to provide appropriate digital infrastructure for teaching and learning [56]. Medical education must adapt to many new and different healthcare contexts, including digitalized healthcare systems and the world of artificial intelligence. The educational design needs to be adapted to the target learners, setting, and available resources [56]. To achieve these goals, the health education industry should prepare the necessary electronic infrastructure and revive its policy regarding the use of digital learning. Additionally, IT technicians and researchers must enhance their use of online tools and databases [20]. To overcome educational challenges and maximize opportunities, effective planning, scientific management, decisive leadership, unified command, and strategic use of technology are essential. Additionally, developing coping mechanisms and providing comprehensive support to higher education institutions impacted by the COVID-19 pandemic are crucial to ensuring the continuity of the education process. To better equip future clinicians to practice during a pandemic, it would be beneficial for them to learn how to adapt to various situations, including a pandemic [21, 57].

After gathering feedback from nursing students about their online learning experiences, it is evident that a blend of virtual and in-person education is preferred, despite a few negative experiences. This valuable insight can be utilized in the development of online education policies for universities in the medical sciences. Given that this research generally highlights the problems of virtual education, specifically in the nursing discipline, future studies could compare problems in online and offline medical education or investigate the effect of blended learning on students' performance. Advancements in educational technologies can enhance the quality and efficiency of virtual education. As educational technology advances, addressing virtual education obstacles is a top priority for enhancing student efficiency and improving learning outcomes.

The present study is limited in its generalizability due to the nature of qualitative studies and differences in nursing students' learning challenges at different universities based on their culture and context. Further research should be conducted on students in different fields at other universities. It is suggested that other researchers conduct mixed methods studies in the field of virtual education. It is possible that some participants were not honest in their answers for several reasons. To gain their trust, the researcher provided sufficient explanations. Researchers used bracketing to prevent the effects of mental preconceptions on the data analysis.

## 5. Conclusion

During the COVID-19 pandemic, undergraduate nursing students experienced both positive and negative aspects of virtual education. While the pandemic has brought many challenges to education, virtual education can be valuable if it is implemented effectively. This study suggested that managers and officials should consider these findings to improve the quality of nursing education.

## Figures and Tables

**Table 1:** Some meaning units, primary codes, and subcategories of the first main category, entitled “positive experiences.”

Main category	Subcategories	Sub-subcategories	Primary codes	Meaning units
Positive experiences	Benefits of virtual learning	Saving time	(i) Timely start and end of online classes (ii) Faster teaching by all teachers in online classes (iii) Elimination of traffic times and commuting from home to university	“*In the online class, if something special happens to the internet or the online system of the university, the online classes start and end on time. What is interesting is that all teachers teach faster in online classes*.” “*I used to go to university 3 times a week; in fact, I'm on the road for 4 hours a week to get from home to school or vice versa. There is always traffic, but since the virtual classes are started, the total time for these courses could be 4 hours, which is great*”
		Saving costs	(i) The financial hurt of the COVID-19 pandemic on everyone except students (ii) Saving the cost of commuting to the university (iii) Saving money due to not having to eat in college (iv) Saving money due to no need to copy professors' handouts (v) Saving the money spent on being with friends	*“The COVID-19 pandemic has hurt everyone financially, except students. Going to college, ordering food weekly, copying professors' handouts, and spending money with friends. All of this has been taken away by COVID-19”*
		Increasing the possibility of daily planning	(i) Having time to do what you want to do (ii) Having motivation (iii) Setting a daily schedule (iv) Practicing the desired program among online classes	*“During this pandemic, I have been able to practice guitar again. I just had the time and motivation and adjusted my schedule during the day to practice between online classes. It makes me feel better”*

## Data Availability

The dataset generated in this study is available from the corresponding author upon reasonable request.
